# Platinum and Gold Blocks

**Published:** 1883-03

**Authors:** 


Platinum and Gold Blocks.—For packing into the interstices about gold plates before soldering, we have never found anything to equal the gold and platina blocks sometimes used in filling teeth. The are of just the proper rigidity to maintain their position during the heating process, and solder flows over them very readily. They may indeed be so manipulated as to make it run almost anywhere one would have it go. If wedged into a crevice closely, they will form a bridge over which the solder may be smoothly conducted; while, if they be loosely packed, it will sink into them as into a sponge, making a solid mass. We have found them very useful in soldering on the porcelain teeth of artificial crowns. In attaching together any two pieces of gold which are not exactly in coaptation, a piece of a platinum and gold block may be inserted to make a bridge for the solder. We have utilized them in mending a hole carelessly melted in a gold plate. In fact, they form a very useful, and to us, a necessary adjunct to the laboratory.

